# Climate Change and Childhood Respiratory Health: A Call to Action for Paediatricians

**DOI:** 10.3390/ijerph17155344

**Published:** 2020-07-24

**Authors:** Maria Elisa Di Cicco, Giuliana Ferrante, Doriana Amato, Antonino Capizzi, Carlo De Pieri, Valentina Agnese Ferraro, Maria Furno, Valentina Tranchino, Stefania La Grutta

**Affiliations:** 1 Department of Paediatrics, University Hospital of Pisa, via Roma 67, 56126 Pisa, Italy; maria.dicicco@unipi.it; 2 Department of Health Promotion, Mother and Child Care, Internal Medicine and Medical Specialties, University of Palermo, Piazza delle Cliniche 2, 90127 Palermo, Italy; 3 Pediatric Medicine Unit and Pediatric Emergency Department, Pediatric Hospital Giovanni XXIII, via Giovanni Amendola 207, 70123 Bari, Italy; dorianamato@libero.it (D.A.); valentina.tranchino@libero.it (V.T.); 4 Pediatrics Unit, S. Paolo and S. Corona Hospital, via Genova 30, 17100 Savona, Italy; anto.capizzi@virgilio.it (A.C.); 2m287@tiscali.it (M.F.); 5 Pediatrics Clinic, Department of Medicine, University Hospital of Udine, Piazzale S.M. della Misericordia 15, 33100 Udine, Italy; carlodepieri@gmail.com; 6 Unit of Pediatric Allergy and Respiratory Medicine, Department of Women’s and Children’s Health, University of Padova, via Nicolò Giustiniani 2, 35128 Padova, Italy; ferrarovalentina@hotmail.com; 7 National Research Council of Italy, Institute for Research and Biomedical Innovation, IRIB, Via Ugo La Malfa 153, 90146 Palermo, Italy; stefania.lagrutta@irib.cnr.it

**Keywords:** allergic rhinitis, asthma, children, climate change, paediatricians, respiratory infections

## Abstract

Climate change (CC) is one of the main contributors to health emergencies worldwide. CC appears to be closely interrelated with air pollution, as some pollutants like carbon dioxide (CO_2_), nitrogen oxides (NOx) and black carbon are naturally occurring greenhouse gases. Air pollution may enhance the allergenicity of some plants and, also, has an adverse effect on respiratory health. Children are a uniquely vulnerable group that suffers disproportionately from CC burden. The increasing global warming related to CC has a big impact on plants’ lifecycles, with earlier and longer pollen seasons, as well as higher pollen production, putting children affected by asthma and allergic rhinitis at risk for exacerbations. Extreme weather events may play a role too, not only in the exacerbations of allergic respiratory diseases but, also, in favouring respiratory infections. Even though paediatricians are already seeing the impacts of CC on their patients, their knowledge about CC-related health outcomes with specific regards to children’s respiratory health is incomplete. This advocates for paediatricians’ increased awareness and a better understanding of the CC impact on children’s respiratory health. Having a special responsibility for children, paediatricians should actively be involved in policies aimed to protect the next generation from CC-related adverse health effects. Hence, there is an urgent need for them to take action and successfully educate families about CC issues. This paper aims at reviewing the evidence of CC-related environmental factors such as temperature, humidity, rainfall and extreme events on respiratory allergic diseases and respiratory infections in children and proposing specific actionable items for paediatricians to deal with CC-related health issues in their clinical practice.

## 1. Introduction

Climate change (CC) is a long-term shift in weather conditions identified by changes in the temperature, precipitation, winds and also including extreme weather events. Such events have been defined as “discrete episodes of extreme weather or unusual climate conditions, often associated with deleterious impacts on society or natural systems, defined using some metric to characterise either the meteorological characteristics of the event or the consequent impacts” [1]. Human activity is the main cause of CC, driven primarily by emissions of carbon dioxide (CO_2_) and enhanced by emissions of other greenhouse gases (GHG). The main anthropogenic contributions to the rising GHG levels include burning fossil fuel, livestock farming, industrial activity and deforestation. CC involves all regions worldwide, with environmental outcomes such as melting of the Polar ice, sea rising and extreme weather events, like heatwaves and drought. CC is one of the main contributors to health emergencies worldwide, with a significant impact on human health and potential long-term consequences. Due to CC, 20–40% of the global human population live in regions that, by the decade 2006–2015, have experienced a warming of more than 1.5 °C above the period of 1850–1900 in at least one season [2]. One of the most relevant goals of the 2030 Agenda for Sustainable Development is to assure health and well-being for all at all ages. However, this goal cannot be reached without addressing the major health determinants that feature in other goals—for example, taking urgent action to combat CC [3].

In March 2020, the British Thoracic Society released the position statement, “The Environment and Lung Health 2020” [4], warning on the upcoming risks of people with chronic respiratory diseases who may be particularly sensitive to the impacts of CC.

In this regard, children, particularly under five years of age, are a uniquely vulnerable group that disproportionately suffers from these adverse impacts [5,6,7,8,9]. Children are more susceptible than adults to CC-related respiratory morbidity due to higher ventilation rates, developing respiratory and immunological systems and smaller peripheral airways [10]. Moreover, they usually spend more time playing outdoors during the warm season, thus being at higher risk of dangerous exposure to high temperatures, given their poor ability to maintain optimal internal temperatures under heat stress [11]. As a dramatic consequence, a child born today will live in a world more than four degrees warmer than the preindustrial average, with CC impacting on their health from infancy throughout life [12].

Even though paediatricians are already seeing the impacts of CC on their patients, their knowledge about CC-related health outcomes with specific regards to children respiratory health is incomplete [13]. This advocates for paediatricians’ increased awareness and a better understanding of the CC impact on children’s respiratory health through integrating evidence into clinical practice and, also, in view of their responsibility to educate families about CC.

This paper aims at reviewing evidence of the burden of CC-related environmental factors such as temperature, humidity, rainfall and extreme events on respiratory allergic diseases and respiratory infections in children and proposing specific actionable items for paediatricians to deal with CC-related health issues in their clinical practice.

## 2. Methods

We searched original papers in English in the PubMed, Scopus and Google Scholar databases using the following keywords, used separately and in combination: Asthma, allergic rhinitis, rhinitis, infections, bronchitis, pneumonia, climate change, pollution, particulate matter, ozone, nitrogen dioxide, allergen and pollen. Age restrictions were set to childhood (birth-18 years). No limitations were set for the date and study country. We also consulted the World Health Organization (WHO) and the Intergovernmental Panel on Climate Change (IPCC) websites and searched the reference lists of the retrieved articles. Exclusion criteria included: commentaries, letters, repeated published articles and case reports.

## 3. Climate Change, Temperature, Pollutants and Pollens: A Harmful Interaction

CC appears to be closely interrelated with air pollution. In fact, some pollutants mainly derived by anthropogenic sources like CO_2_ are naturally occurring GHG that contribute to global warming. High temperatures accelerate the production of ozone (O_3_) from its precursors—volatile organic compounds (VOCs), carbon monoxide (CO) and nitrogen oxides (NOx)—so that high levels of this pollutant are concerning large areas of the world. On the other hand, the concentrations of pollutants such as fine particulate matter (PM), polycyclic aromatic hydrocarbons (PAHs) and black carbon are expected to rise due to changes in the temperature, precipitation frequency and forest fires induced by CC [14]. An overabundance of GHG traps a large amount of heat in the atmosphere by absorbing the sun’s energy and redirecting it back to the earth’s surface, thereby accounting for global warming [15].

High temperatures due to global warming are estimated to have an influence on the start and duration of plant pollen production; as a matter of fact, the increase in fall temperatures results in earlier flowering for many spring-flowering species around the world. Recently, a retrospective analysis of global datasets, with 20 years or more of airborne pollen data in 17 locations in the Northern hemisphere, revealed the significant increase of the pollen load and pollen season duration over time in most analysed locations, in association with the annual cumulative increases in temperature over time. Since air pollen concentrations are supposed to reflect the spatial and temporal changes of plant species, these results suggest that CC, through high temperatures, is already affecting season timing and duration, thereby increasing pollen amounts in the Northern hemisphere [16]. Nonetheless, airborne pollen evolution depends also on pollen production, release and atmospheric dispersion changes. With regards to the European region, it was estimated that, by 2050, airborne ragweed pollen concentrations will be about four times higher than now, mainly due to climate and land-use changes that will extend the ragweed habitat suitability in Northern and Eastern Europe and increase the pollen production in established ragweed areas owing to the increasing CO_2_ [17].

The interaction between air pollution and aeroallergens has been demonstrated in studies showing that pollutants are able to increase pollen production [18] and to modify pollen morphology and content [19], as well as the chemotactic and the immunomodulatory properties [20]. Open-field studies confirmed these findings. Using an existing temperature/CO_2_ gradient between urban and rural areas, Ziska et al. showed that, the average daily values of CO_2_ concentrations and air temperatures within an urban environment were 30% to 31% and 1.8 degrees to 2 °C higher than those at a rural site. Examining the quantitative and qualitative aspects of ragweed growth and pollen production, they found that ragweed grew faster, flowered earlier and produced significantly greater aboveground biomass and pollen [21].

Interestingly, some studies demonstrated that pollen grains can absorb heavy metals but, also, nitrate and sulphur, while others showed that particles can agglomerate on the surface of pollen grains [22,23,24]. This pollen-particle interaction may modulate the allergen release and the absorption of pollen proteins to airborne particles, finally contributing to the increase in pollen allergies and asthma in highly polluted areas [19].

**Figure 1:**
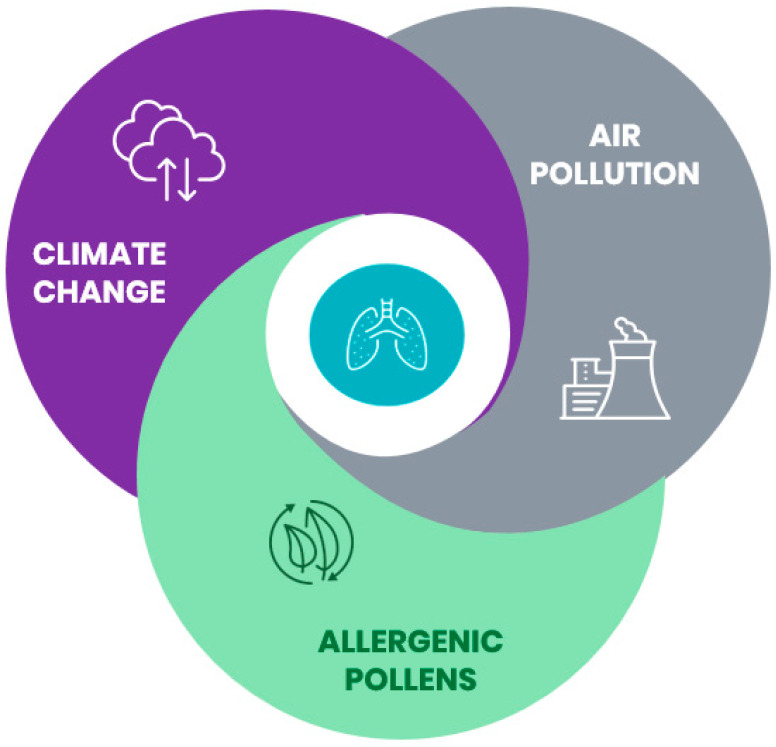
Climate change, air pollution and allergenic pollens are closely interrelated and may impact respiratory health.

Taken together, this evidence is suggestive for a complex interplay amongst CC, air pollution and allergenic pollens that may have a deep impact on respiratory health (Figure 1).

## 4. The Impact of Climate Change on Paediatric Respiratory Allergic Diseases

### 4.1. Temperature

With regards to the prevalence of allergic rhinitis (AR), a global ecological analysis in adolescents (222 centres in 94 countries) and in children (132 centres in 57 countries), who had taken part in phase III of the ISAAC (International Studies of Asthma and Allergies in Childhood) study, showed several spatial associations between climatic factors and the prevalence of intermittent and persistent AR symptoms. Monthly data on rhinitis symptoms were collected via standardised parent-completed (for children) or self-completed (for adolescents) questionnaires investigating if problems with sneezing or a runny or blocked nose, eventually accompanied by itchy eyes, apart from a cold or flu, had occurred in the last 12 months. In adolescents, between-country significant associations were found for intermittent rhinitis prevalence and the mean and minimum temperatures (*p* < 0.01); significant within-country associations were found, especially for persistent rhinitis prevalence and the mean and maximum temperatures (*p* < 0.05). Overall, these results indicate that the temperature may contribute to the global increased prevalence of rhinitis symptoms, likely altering the timing, spatial distribution, quality, and quantity of allergenic plants and pollens [25].

A more recent study conducted in China on adults and children showed that pollen-induced AR symptoms were positively correlated with temperature (*p* < 0.05), which, in turn, was positively correlated with the pollen counts (*p* < 0.001), suggesting that a pollen-induced AR prevalence depended on seasonal pollen exposure, which was influenced by local climate factors [26].

With regards to asthma, an ecological study in Australia showed that both cold and hot temperatures may influence the risk of access to emergency care for asthma. In particular, male children and those aged ≤4 years were more susceptible to heat; male children and those aged 10–14 were more susceptible to cold, emphasising the damaging influence of extreme temperatures on the risk of asthma attacks in children [27].

More recently, a study reported lagged effects for temperature-related and paediatric asthma emergency visits, suggesting that children are at risk for increased asthma morbidity when temperatures are high, on average, for several days. In detail, estimated Relative Risks (RRs) for maximum temperatures and asthma emergency visits were positive and significant for lag days 1–5, with the strongest single-day association observed on lag day 2 (RR = 1.06, 95% CI: 1.03, 1.09) for a change in the T_max_ from 27 °C to 32 °C (25th to 75th percentile) [28].

### 4.2. Humidity

In a previous Turkish study on children, AR was significantly associated with a higher mean yearly outdoor humidity (more than 70%) (*p* < 0.05) [29]. More recently, the 2007 U.S. National Survey of Children’s Health reported that the prevalence of hay fever in children was significantly lower with the second and third quartile mean annual relative humidity (*p* ≤ 0.01), fourth quartile mean annual Palmer Hydrological Drought Index (a lower score reflects dryer conditions, whereas a higher score reflects wetter conditions) (*p* = 0.02) and third and fourth quartile mean annual heating degree days (*p* < 0.0001) but increased with the second, third and fourth quartile mean annual temperatures (*p* ≤ 0.02) and the fourth quartile mean annual precipitation (*p* = 0.0007) [30].

With respect to paediatric asthma, inconsistent results were found, possibly due to the heterogeneity of the study designs and asthma outcomes (morbidity, exacerbations and emergency visits), as well as differences in the geographical environments, type of climates, vegetation, air quality and socioeconomic conditions [6]. A Taiwanese study reported that winter humidity was positively associated with the prevalence of asthma in middle-school students (girls: *p* < 0.05 and boys: *p* < 0.01). [31]. A positive relationship between the mean monthly relative humidity and childhood asthma was reported in a Greek study, where a 10% increase in humidity was related to a 31% increase in the probability of having an admission for asthma [32].

Contrariwise, relative humidity was negatively correlated with the asthma admission rates of pre-school (*p* < 0.01) and school-age children (*p* < 0.001) in a Taiwanese study [33]. A population-based study in Spain also showed that relative humidity was negatively associated with asthma in children aged 13 and 14 years (*p* < 0.0001) [34].

### 4.3. Extreme Weather Events

Very recently, scientific interest has been focused on the impact that extreme events may have on childhood asthma. Climatic indices such as the Atlantic multidecadal oscillation (AMO) and the Pacific decadal oscillation (PDO), which are known to modulate drought periods in the contiguous United States, have been positively correlated with asthma death rates in children aged 5–14 years over the period from 1950 to 2015, proving that drought variations and climate variability may be emerging risk factors for asthma mortality [35].

A time-stratified case-crossover study examined the association between the exposure to extreme precipitation events and risk of hospitalisation for asthma in Maryland (USA) between 2000 and 2012: exposures to extreme precipitation, especially during summer, were associated with an increased risk of hospitalisation for asthma. In detail, a higher risk was observed among children aged ≤4 years (OR: 1.20, 95% CI: 1.05, 1.37) and 5 to 17 years (OR: 1.11, 95% CI: 0.94, 1.30) [36].

Evidence is also growing for the associations between the occurrence of thunderstorms and asthma exacerbations. The so-called “thunderstorm asthma” indicates the bronchospasm developing after a storm during the pollen season, when the pollen grains concentrate at the ground level and, following their rupture due to osmotic shock, they release allergenic particles that can be inhaled, favouring the exacerbation of asthma in people allergic to pollens [37]. An ecological time series analysis conducted for asthma presentations to emergency departments (ED) among children in Melbourne, Australia reported that, during thunderstorms, the daily ED attendance peaked at 70 visits per day, consistent with thunderstorm-associated asthma related to the preceding extreme grass pollen days and strong winds [38]. Nonetheless, it should be pointed out that the risk of asthma exacerbations due to extreme events like thunderstorms in children needs further exploration in order to elucidate the role of concomitant factors, such as viral infections and atopy, in eliciting or worsening symptoms.

### 4.4. Air Pollution

The complex interplay amongst CC, air pollution and allergenic pollens may contribute to the damage of the airway mucosa through impaired mucociliary clearance, increased permeability with the consequent facilitated penetration of allergens and stimulation of the immune response with an increased production of Immunoglobulins (IgE). Changes in the temperature, precipitation frequency and air stagnation due to CC, as well as forest fires, may release large amounts of PM, PAH and black carbon in the air, which may affect large numbers of exposed children, leading to adverse respiratory outcomes, such as asthma exacerbations [14].

The relative impact of climatic variables and air pollutants on childhood respiratory allergic diseases is an emerging field of research. Noteworthy, a very recent study aimed to quantify the relative impact of meteorological factors and air pollutants on childhood allergic diseases in China showed that AR and asthma prevalence were inversely associated with the daily mean temperature and positively associated with the daily air pressure, NO_2_ and O_3_. However, the climatic factors appeared to play a more relevant role than air pollutants. Indeed, the number of diseases attributable to an interquartile range change in climatic factors was greater than those attributable to an interquartile range change in pollutants, emphasising the higher burden of childhood AR and asthma related to climatic variables [39].

Taken together, these data provide evidence of the relevant role of CC-related meteorological conditions on the prevalence of childhood AR, as well as on asthma prevalence and morbidity (Table 1). Of note, an additional impact by air pollutants has been reported on both AR and asthma morbidity.

## 5. The Impact of Climate Change on Paediatric Respiratory Infectious Diseases

The impact of CC on respiratory infectious diseases has recently raised an increasing research interest, particularly in children affected by chronic respiratory diseases [4].

### 5.1. Temperature

In children under five years of age living in the tropical area of Mekong River Delta, through multivariate distributed lag models, it was shown that a 1 °C increase in the average temperature of the previous day and the previous two days was associated with a 1.9% (95% CI 0.3, 3.4) increase in hospital admissions for respiratory infections; when the cumulative lag model was considered, such an increase was larger: 3.8% (95% CI 0.4, 7.2). To explain these findings, the authors proposed the two following reasons: first, younger children do not practice as much outdoor activity as the older ones, and second, parents may decide not to take the younger children out when the outdoor temperature is increasing [40].

Both the temperature variability, commonly defined as the temperature change between two neighbouring days (TCN), and the difference between the daily maximum temperature and the daily minimum temperature within one day (DTR) have been recognised as a risk factor for respiratory infections, too. Xu et al. previously reported that a TCN ≤ 2 °C may increase the risk of emergency visits for childhood pneumonia in Brisbane, Australia; this effect lasted around three weeks. Female children, Indigenous children and children aged 5–14 years were the most vulnerable [41]. Moreover, a study in China reported that the DTR was significantly associated with acute bronchitis (defined as an inflammation of the large bronchi mainly characterised by cough, ICD-10 codes: J20) in children, especially when it exceeded 10.9 °C; the largest effect was shown at a three-day lag, with a 1% (95% CI: 0.5, 1.6) increase of acute bronchitis cases per 1 °C increment of the DTR. Female children and children aged 0–4 years were the most vulnerable [42].

Interestingly, a recent study from South Korea revealed that a “pneumonia temperature”, i.e., day-to-day variance in temperature, was associated with an increased risk of emergency visits for pneumonia. In children aged 0–5 years, when the pneumonia temperature was higher than 6 °C, each additional 1 °C increase resulted in a 1.89% (95% CI: 1.34, 2.67) increase in the relative risk for an emergency visit due to pneumonia. Warm average temperatures and large DTRs showed protective effects, even if the latter might have been influenced by some limitations, such as not considering the effect of modifiers like the season and socioeconomic status [43].

### 5.2. Humidity

Of note, the burden of higher temperatures seems to be counterweighted by an increased humidity; in addition, the activity of many respiratory viruses, such as respiratory syncytial virus (RSV), influenza A and rhinovirus, is positively correlated to the relative and/or absolute humidity [44,45,46]. In the Netherlands, during the period 1998–2005, the relative humidity was positively associated with RSV activity for up to four weeks (*p* < 0.01), suggesting more RSV activity when the relative humidity increased and, therefore, supporting the role of climatic conditions in the dynamics of RSV infection [45]. Accordingly, in a study conducted in an urban area in Italy, the number of RSV-positive infants was correlated with the relative humidity (*p* < 0.001) positively and with temperature (*p* < 0.001) negatively [47].

### 5.3. Extreme Weather Events

An effect of CC seems to be the higher frequency of rainfall events, with the consequence of a larger proportion of people being exposed to pluvial floodwater in both high and low-income regions. On the other hand, urbanisation also contributes to the increased risk of flooding through relevant changes of the land cover.

Potential adverse health impacts of floods include respiratory infections. An increased risk of 1.66 (95% CI: 1.57, 1.74) for hospital admissions due to acute lower respiratory infections in children aged 0–15 years has been reported in the Mekong River Delta area [48]. Extreme rainfall was significantly associated with emergency visits for influenza in children aged 5–18 years (OR = 1.32 (95% CI: 1.14, 1.52)) in the North-eastern U.S. area, too. Notwithstanding the strict methodology of this case-crossover study, including climatological and air pollutants, temperature, relative humidity and barometric pressure in the analysis as potential confounders, the authors recognise that further studies are required to clarify what could be the role of indoor crowding in this association [49]. More recently, a cross-sectional survey in 60 Dutch locations with reported flooding did not find a significant association between the exposure to pluvial floodwater and acute respiratory infections in children (OR = 1.6 (95% CI: 0.6, 4.0)) [50].

In a Brazilian study, the combination of rainfall, air temperature, humidity and wind speed showed relatively high correlation coefficients in relation to hospital admissions for pneumonia in children (R_2_ = 68.4%) and infants (R_2_ = 71.8%) [51].

Extreme weather events such as heatwaves have become one of the most serious consequences of CC, which may promote or aggravate respiratory diseases. In California, during heatwaves, emergency department visits for acute respiratory infections increased among children aged 0–4 years (RR: 1.05; 95% CI: 1.04, 1.07) [11]. More recently, an ecological study in China in the period 2010–2012—during which four heatwaves occurred—found significant positive associations of respiratory infectious diseases with heatwaves. In particular, in children aged 4–17 years, the strongest effect was shown at lag 0 (RR = 1.764; 95% CI: 1.479, 2.103) for upper respiratory tract infections. The results were adjusted for meteorological (temperature, rainfall, wind speed, pressure, humidity and sunshine hours) and air pollution factors, including the level of several atmospheric pollutants (nitrogen oxides, sulphur dioxide, total suspended particulates, etc.) [52].

Taken together, these data provide evidence of the role of CC-related factors such as high temperatures, temperature variability, humidity and extreme weather events on respiratory infection morbidities in children.

## 6. The Role of Paediatricians: A Call to Action

Since CC may undermine the last half-century of gains in global child health [5,13], there is an urgent need for health professionals to successfully educate citizens about CC issues and to engage them in preventive and adaptive responses [53]. 

In order to minimise CC effects on respiratory health, physicians should consider measures such as learning more about CC health impacts, understanding pollen and air quality forecasts, teaching patients and their families to find available pollen data and counselling caregivers to minimise exposures and maximise the management of the respiratory symptoms of their children [54,55], as well as using anticipatory guidance to discuss CC with families and serving as a personal role model [56].

Paediatricians, who bear a special responsibility for children, should be engaged in strategies to address CC by alerting patients and their families on health implications and, also, counselling on the personal and environmental benefits of a transition to active transport and to a plant-based diet [57,58,59,60]. Indeed, consuming healthy diets low in red meat and processed meats and rich in plant-based foods has been suggested to substantially contribute to reducing CO_2_ emissions, crop damage and extreme weather [61]. However, interventions to address CC encompass not only behavioural changes but, also, technological advances and political action. Paediatricians should exert pressure on governments, corporations and private institutions to take mitigation strategies for CC. They also should be involved in continuing medical education activities within teaching modules, as well as residency training programmes and conferences, in order to strengthen their leadership capacity in the area of children’s environmental health [57].

Suggested actions for paediatricians to improve their daily clinical practice are summarised below.

- Refer to existing resources that provide climate-smart healthcare practices (e.g., American Academy of Pediatrics policy statement on global CC and children’s health, http://pediatrics.aappublications.org/content/136/5/992).
- Teach parents and caregivers to minimise their own carbon footprint (e.g., Centers for Disease Control and Prevention. Sustainable Lifestyle, https://www.cdc.gov/sustainability/lifestyle/index.htm and United States Environmental Protection Agency Carbon Footprint Calculator, https://www3.epa.gov/carbon-footprint-calculator/).
- Counsel patients to monitor the local information on weather, heat waves, air quality and pollen forecasts in order to set preventive measures and developing corrective behaviours for minimising adverse environmental exposures (e.g., maintain regular controller treatment for symptom control, carry a rescue inhaler on high-risk days, limit time outdoors and avoid intense or prolonged exertions outdoors).
- Modify the anticipatory guidance for CC-related impacts, especially for children with chronic respiratory diseases (e.g., adequate hydration and cooling on hot days and advice for vulnerable individuals to avoid intense outdoor exercise and stay indoors or wear protective masks when the air quality is in the harmful range).
- Teach patients to recognise respiratory symptoms that may be related to extreme weather events (e.g., rhinorrhoea, sneezing, itching, cough, phlegm, wheezing, shortness of breath and chest tightness).
- Counsel parents and caregivers on the personal and environmental benefits of transitioning to active transport (e.g., walking, jogging and bicycling) and a plant-based diet (e.g., Mediterranean diet), as consuming diets low in red meats and processed meats and rich in plant-based foods has been suggested to substantially contribute to reducing CO_2_ emissions, crop damage and extreme weather.

Paediatricians should also play a central role at an institutional level in the global challenge imposed by CC by helping to build a broader coalition across disciplines to properly address CC [60,62]; advocating for local, national, and international policies aimed at reducing GHG emissions and taking part in multidisciplinary collaborations with other health professionals, civil authorities in charge of emergency preparedness programmes, city planners and architects, in order to develop new adaptation measures and strengthen existing public health strategies [63,64].

## 7. Conclusions

CC is currently one of the most dangerous threats to children’s health. Children are uniquely vulnerable to CC, due to age-specific characteristics. There has been increasing interest in CC’s impact on their respiratory health.

Paediatricians can play a relevant role in a range of actions, which include implementing and promoting strategies to reduce GHG and providing education to children and their caregivers but, also, being involved in policy work. In this context, they should collaborate with decision-makers, including local governments, academia and school boards and community leaders. Paediatricians can also conduct health assessments, actively support policies to reduce harmful exposures and advocate for protecting children’s health [65]. Such actionable items should be acknowledged by paediatricians to fully address CC-related health issues in their clinical practice, with the aim of reducing the risks and improving the health of children.

## Figures and Tables

**Table 1:** The impact of climate change (CC)-related meteorological conditions on allergic rhinitis (AR) and asthma in children.

CC-Related Meteorological Conditions	AR	Asthma
**Temperature**	Prevalence of AR symptoms is positively associated with temperature [25,26].	Cold and hot temperatures influence the risk of access to emergency care [27,28].
**Humidity**	Inconsistent association with AR prevalence [29,30].	Inconsistent association with asthma prevalence and outcomes [6,31,32,33,34].
**Drought**	Prevalence of AR is lower in areas with a higher drought index (wetter conditions) [30].	Drought variations are positively correlated with asthma death rates [35].
**Rainfall**	Minimum and maximum precipitation are positively associated with AR symptom prevalence [25].	Exposures to extreme precipitation are associated with an increased risk of asthma hospitalisation [36] and epidemics of thunderstorm asthma [38].
